# Hepatoprotective Effect of Supercritical Carbon Dioxide Extracted Dabai Pulp Oil and Its Defatted Pulp

**DOI:** 10.3390/molecules26030671

**Published:** 2021-01-28

**Authors:** Noor Atiqah Aizan Abdul Kadir, Azrina Azlan, Faridah Abas, Intan Safinar Ismail

**Affiliations:** 1 Department of Nutrition, Faculty of Medicine and Health Sciences, Universiti Putra Malaysia, Serdang 43400, Selangor, Malaysia; atiqahaizan@yahoo.com; 2 Research Centre for Excellence for Nutrition and Non-Communicable Disease, Faculty of Medicine and Health Sciences, Universiti Putra Malaysia, Serdang 43400, Selangor, Malaysia; 3 Halal Products Research Institute, Universiti Putra Malaysia, Serdang 43400, Selangor, Malaysia; 4 Department of Food Science, Faculty of Food Science and Technology, Universiti Putra Malaysia, Serdang 43400, Selangor, Malaysia; faridah_abas@upm.edu.my; 5 Department of Chemistry, Faculty of Science, Universiti Putra Malaysia, Serdang 43400, Selangor, Malaysia; safinar@upm.edu.my

**Keywords:** antioxidant, functional food, hepatoprotective, hypercholesterolemia, waste

## Abstract

All food scientists must utilize plants for their application as functional foods to reduce hypercholesterolemia incidence through diet. *Canarium odontophyllum* (dabai) is a novel source for new healthy oil and functional foods. In this work, we evaluate the hepatoprotective effects of supercritical carbon dioxide (SC-CO_2_) extracted dabai pulp oil (DPO) and defatted dabai pulp (DDP) against hypercholesterolemia elicited by a high-cholesterol diet in rats. Our results show that DPO and DDP supplementation exerted beneficial hypocholesterolemic effects against the high-cholesterol diet-fed rat. Nevertheless, supplementation with DDP revealed superior total cholesterol, low-density lipoprotein, and HMG-CoA reductase lowering efficacy (*p* < 0.05). Supplementation of either DPO or DDP did not significantly affect AST and ALT levels than normal rats (*p* > 0.05). Therefore, DDP and DPO are considered as having no toxicological significance. The histological section of rats treated with DPO and DDP showed improved steatosis in hepatocytes. HPLC analysis revealed that DPO and DDP contained syringic acid, which plays an important role in the beneficial effect. In conclusion, our results support the hypocholesterolemic and hepatoprotective effects of DPO and DDP in the hypercholesterolemic rats model.

## 1. Introduction

*Canarium odontophyllum* or “dabai” is an indigenous seasonal fruit that can only be found in Borneo Island, especially in the Sibu and Kapit of Sarawak, Malaysia. Dabai fruit is recognized as Sibu olive because of its similar appearance, flavor, and texture with olive [1]. Interestingly, the dabai fruit fractions (pulp and seed) possess various biological activities such as antimicrobial (seed) [2], anti-fungal (pulp) [3], anti-Alzheimer’s (pulp and seed) [4], and anti-hyperglycemic (pulp) [5]. Phytochemicals identified from dabai fruit (pulp with peel), were phenolic acids (ellagic, vanillic acids, ethyl gallate), flavonoids (catechin, epicatechin, epicatechin gallate, epigallocatechin gallate, apigenin), anthocyanins (malvidin-3,5-di-*O*-glucoside, cyanidin-3-*O*-glucoside, cyanidin-3-*O*-rutinoside and peonidin-3-*O*-glucoside), and anthocyanidins (cyanidin, pelargonidin and delphinidin) [6]. Meanwhile, carotenoids such as all-*trans*-β-carotene, 15-*cis*-β-carotene, 13-*cis*-β-carotenes, 9-*cis*-β-carotene, di-*cis*-β-carotene, all-*trans*-lutein, 9-*cis*-lutein, 13-*cis*-lutein were found in the peel, pulp and seed of dabai fruit extracts [7].

In the past studies, supplementation of chloroform-methanol extracted dabai pulp oil (DPO) resulted in favorable changes in blood lipid (increased in high-density lipoprotein and reduce in low-density lipoprotein) and lipid peroxidation with the enhancement of superoxide dismutase (SOD), glutathione peroxide (GPx), and plasma total antioxidant status (TAS) levels in healthy rabbits [8]. Meanwhile, supplementation of chloroform-methanol extracted defatted dabai pulp (DDP) in hypercholesterolemic rabbits showed cholesterol-lowering effect (reduced plasma low-density lipoprotein and total cholesterol levels) as well as reduced atherosclerotic plaques [9] and possessed cardioprotective effects [10]. Although previous findings of solvent extracted DPO and DDP concluded that both offer health potential in the studied rabbits, the conventional chloroform-methanol extraction could be toxic to humans because of the potential leftover of solvent residue in the extracted oil and the defatted part.

Consumer interest in functional foods has increased dramatically because of the rising awareness of the health benefits of the consumption of foods enriched in bioactive compounds [11]. Antioxidants that even exist at lower concentration can significantly delay or even prevent oxidation of oxidizable substrates [12]. Antioxidant shows therapeutic activities in various diseases such as cardiovascular diseases, cancer, and liver diseases [13,14]. Specifically, syringic acid (4-hydroxy-3,5-dimethoxybenzoic acid, SA) was known to possess antioxidant [15], cardioprotective [16], hepatoprotective [17], and anti-steatotic activities [18].

Food or plant-derived extracts are the sources for the formulation of functional foods. Several extraction techniques can obtain functional food rich in bioactive compounds [19]. However, food scientists should consider the toxicity of the solvents used, the degradation of compounds, and the process’s selectivity in the manufacturing process [20]. The conventional extraction procedure such as solvent extraction, showed some disadvantages, such as time-consuming and having low selectivity. Additionally, the use of organic solvents does show possible toxicity and unease about eco-sustainability [21].

Increased preference for food production quality and safety has driven supercritical fluid extraction (SFE) to be the primary alternative option for the sample preparation step to either strip the unwanted material from a product or collect the desired product [22]. SFE can be a fast, efficient, and clean method for extracting desired products from vegetables or fruits matrices [23]. Extraction of oil and its waste using SFE is considered a novel green technology because the final product’s characteristics can easily be altered by changing the process parameters [24]. Hence, SFE can be used as an alternative technique to produce a non-toxic DPO and DDP. Production of DPO and DDP by SFE is still relatively new in Malaysia. This study evaluates the hepatoprotective effect of supercritical carbon dioxide (SC-CO_2_)-extracted DPO and DDP elicited by a high-cholesterol diet in rats.

## 2. Results and Discussion

### 2.1. Phenolic Characterization

DPO is a newly extracted oil, and DDP is the waste of supercritical carbon dioxide (SC-CO_2_) extraction, and we expect that DPO and DDP possess polyphenol content. Indeed, the total phenolic content (TPC) in DPO was 0.118 ± 0.01 mg GAE/g extract, while the TPC in DDP was 4.404 ± 0.09 mg GAE/g extract.

The SA in DPO was 2.11 ± 0.03 µg/mL, whereas the SA in DDP was 89.87 ± 15.18 µg/mL. SA is well-known to have hepatoprotective activity. SA exerts hepatoprotective activity on CCl_4_ [25] and concanavalin-A induced chronic liver injury mice model [26]. Moreover, in comparison with silymarin, a standard hepatoprotective drug, SA exhibits potent hepatoprotective effects in acetaminophen-hepatotoxicity-induced rats [27].

As demonstrated in Table 1, the incorporation of DDP in the treatment diet showed a significantly higher total polyphenol than the incorporation of DPO in the treatment diet. Greater SA content in DDP contributes toward higher polyphenol content in the DDP treatment diet. These results provide evidence that DDP still contains the bioactive compound.

In this study, the total anthocyanin content in DDP was determined as 523.3 ± 22.36 mg/100 g. Anthocyanins are mostly found in the peel of purple-colored berries [28]. Interestingly, the dabai pulp peel is also purple. The purple skin of dabai fruit was due to anthocyanin that contributed to the higher polyphenols’ content [29]. Phenolic compounds were reported to show multifunctional properties and beneficial effects on human health. Because of this astonishing finding, polyphenols have become a great interest among food scientists [30]. A high level of phenolic content had been reported in dabai fruit, where the pulp has 267.0 ± 4.24 mg GAE/100 g of total phenolic content [31]. Excellent polyphenol content in DDP corroborates the idea that DDP is plausible to be developed as novel sources of bio-functional ingredients to formulate functional food.

### 2.2. Hypocholesterolemic Effect of DPO and DDP

Table 2 shows the effect of DPO and DDP treatment on serum lipid parameters in rats. There was a significant elevation of total cholesterol (TC), low-density lipoprotein (LDL), and HMG-CoA reductase (HMG-CoA-r) in hypercholesterolemic rats (PG) when compared with normal rats (NG) (*p* < 0.05). Based on these results, we had established hypercholesterolemia in rats using a high cholesterol diet. Treatment with DPO and DDP significantly reduced the TC, TG, and HMG-CoA-r levels compared with the PG group (*p* < 0.05). Further, we also noticed a significant reduction of serum LDL detected in rat fed with DDP compared to the PG group (*p* < 0.05).

Supplementation of 2% DPO and 2% DDP exerted beneficial effects against the high-cholesterol diet-fed rat. Nevertheless, supplementation with 2% DDP resulted in significantly superior serum change of TC, LDL, and HMG-CoA-r than the PG group (Table 2). Similar findings were observed in the study by Shakirin et al. [9]. Further, anthocyanins in DDP are potential factors contributing to hypocholesterolemic rabbits’ cholesterol-lowering effect [9]. Previously, Khoo et al. [29] demonstrated that anthocyanins are the main phenolic compound identify in DDP.

In this study, supplementation of 2% DDP rich in anthocyanin (523.3 ± 22.36 mg/100 g) could explain the hypocholesterolemic effect observed in the rats treated with the 2% DDP group. Hence, the possible mechanism behind the hypocholesterolemic effect of anthocyanins occurs in cholesterol synthesis inhibition. Anthocyanin can stimulate AMP-activated protein kinase (AMPK). AMPK is involved in regulating energy homeostasis and influencing many enzymes, including HMG-CoA reductase [32]. Also known, HMG-CoA reductase is the rate-limiting enzyme in cholesterol synthesis. An increase in AMPK activity would inhibit cholesterol synthesis, which leads to a lower cholesterol level [33].

Additionally, AMPK is also involved in the inhibition of acetyl-CoA carboxylases ACC1 and ACC2, which cause an increase in fatty acid oxidation and a decrease in fatty acid synthesis accordingly, leading to lower triglyceride concentration [34]. There is a possibility that anthocyanin in DDP activates AMPK, which inhibits cholesterol synthesis and leads to lower cholesterol in rats treated with DDP. These demonstrate the novel application of DDP as a functional property in foods.

### 2.3. Evaluation of Hepatic Steatosis

Table 3 shows the liver weights of rats in the various groups. There were no significant alterations in the liver’s weights in the DPO- and DDP-treated groups than normal rats. There were, however, significant increases in the liver weights of the hypercholesterolemic rats compared to those of the normal rats (*p* < 0.05). Organ weight is a sensitive indicator of an experimental compound’s effect, as a significant difference in organ weight detected between treated and untreated animals may indicate any morphological changes [35].

Hypercholesterolemic rats’ liver weight showed significantly higher than normal rats. Meanwhile, rats treated with 2% of DPO, 2% of DDP, and simvastatin showed no liver weight variation compared to normal rats. In this study, feeding a high cholesterol diet caused a change in liver weight. Higher liver weight could be a consequence of higher fat content in the liver [36].

One of the central functions of the liver is to detoxify toxins from the body. Liver function tests were carried out to evaluate hepatocyte injuries and to assess liver function. AST and ALT are the markers of hepatocyte injuries. AST and ALT are introduced to the bloodstream following situations that involved either cell damage or necrosis [37]. Additionally, the transaminase levels show toxicity to the liver or changes in the structure of the liver cells’ membrane. AST is a sensitive but nonspecific indicator of hepatocellular changes as this enzyme can be released from many damaged organs such as the liver, heart muscle, kidney, and pancreas. Meanwhile, ALT is a more specific enzyme for registering liver damage [38]. Serum ALT activity was perceived as a reliable and sensitive marker for liver disease and a reliable marker of the overall health condition [39].

As indicated in Table 3, hypercholesterolemic rats showed significant AST and ALT elevation compared to normal rats. A similar result showed that AST and ALT’s elevation was detected in hypercholesterolemic rats by De Souza et al. [40]. Generally, hypercholesterolemia is associated with toxicity due to elevated liver enzymes and lipids’ peroxidation in serum and tissue [41,42]. There was a significant elevation of AST and ALT seen in rats treated with simvastatin. The most significant adverse effects of statins (atorvastatin, fluvastatin, lovastatin, pravastatin, rosuvastatin, and simvastatin) are asymptomatic increases in liver transaminases and myopathy [43]. On the contrary, supplementation of 2% of DPO and DDP showed no significant AST and ALT variation than the normal group. Therefore, the results indicated that SC-CO_2_ DPO and DDP could be considered as having no toxicological significance.

**Figure 1:**
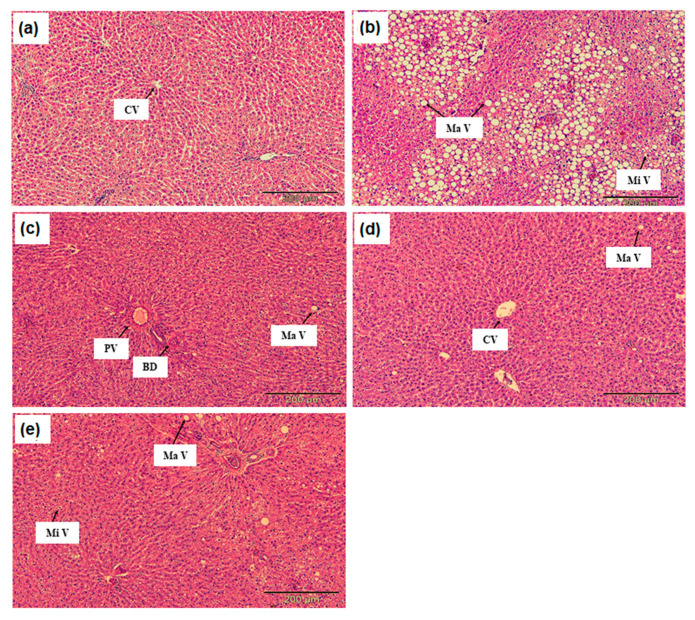
(**a**) Histology section of liver tissue in the normal rat group (NG), (**b**) hypercholesterolemic positive control rats group (PG), (**c**) hypercholesterolemic rats treated with 2% DPO (HG), (**d**) hypercholesterolemic rats treated with 2% DDP (DG) (**e**) hypercholesterolemic rats treated with 10 mg/kg simvastatin (SG) observed under a light microscope at 200× magnification (hematoxylin and eosin staining). CV: central vein; MaV: macrovesicular steatosis; MiV: microvesicular steatosis; PV: portal vein; BD: bile duct.

Figure 1 shows the animals’ liver sections; no histological abnormalities were found in the normal rats’ hepatocytes (Figure 1a). Meanwhile, hypercholesterolemic rats’ liver section showed serious fat vacuoles indicating hypercholesterolemic rats developed a high degree of steatosis induced by a high cholesterol diet (Figure 1b). In this study, liver steatosis was the main histopathological feature observed in the hypercholesterolemic rats’ livers. The macrovesicular steatosis is the predominant pattern. The severity was evaluated based on the following scoring scheme: - normal, + mild effect, ++ moderate effect, +++ severe effect [44].

Steatosis or a fatty liver is a change accumulation of the abnormal amount of lipids in 5% or more hepatic cells. Macrovesicular steatosis is the most common type of steatosis, seen as a single large fat vacuole or several smaller ones occupying the more significant part of the cell, pushing the nucleus to the periphery. On the other hand, microvesicular steatosis is the less common and often more severe steatosis type [45]. The fat in the microvesicular is finely divided, and the nucleus remains central. Sometimes, the two types of steatosis are found together. Microvesicular steatosis happens as a result of mitochondrial damage leading to impaired β-oxidation [46]. Meanwhile, macrovesicular steatosis is made apparent by non-invasive imaging and may be accompanied by moderate abnormalities of serum amino-transferases, alkaline phosphatase, and gamma-glutamyl transpeptidase [47].

This fact agrees with the liver function test of hypercholesterolemic rats, as the ALT and AST were significantly higher than normal rats, indicating the high degree of steatosis. The lipid in macrovesicular steatosis accumulates in hepatocytes because of increased triglyceride synthesis or decreased excretion. This study demonstrated that high triglyceride (Table 2) in hypercholesterolemic rats could explain the severe fat vacuole seen in the liver section.

In contrast, the histological section of rats treated with 2% DPO and 2% DDP showed improved steatosis in hepatocytes (Figure 1c,d). DPO and DDP contained SA (2.11 ± 0.03 µg/mL and 89.87 ± 15.18 µg/mL respectively). SA is a benzoic acid derivative that can be found in edible fruits and plants. Ham et al. [18] demonstrated that SA possesses anti-steatotic effects. Astoundingly, SA ameliorated hepatic steatosis by stimulating fatty acid oxidation via Pparα and suppressing lipid synthesis through Pparγ-Srebp-1c and reduced cholesterol accumulation by inhibiting Srebp-2 [18]. This study demonstrates that DPO and DDP contained SA and shows a hepatoprotective effect and may likely be a therapeutic agent for hypercholesterolemia or non-alcoholic liver disease.

## 3. Materials and Methods

### 3.1. Sample Collection and Preparation

Fresh dabai fruits (226 kg) were collected from Sarikei Sarawak, Malaysia, with the assistance and permission of the Agricultural Research Centre (ARC), city, Sarawak, country. All samples collected were matured fruits. The official identification of the fruits’ variety and maturity was aided by the Agriculture Research Centre (ARC) research officers, Semongok, Sarawak, Malaysia, and the herbarium voucher specimens (S 64872) of these fruits were deposited. Dabai fruits were shipped to the Faculty of Medicine and Health Sciences, Universiti Putra Malaysia, Serdang, Malaysia. Dabai fruits were examined and washed under running tap water, then soaked in warm water (36 °C) for 15 min to soften the pulp, and the seeds were removed. Then, dabai pulp was sent to Phytes Biotek Sdn Bhd for freeze-drying using an industrial scale freeze dryer (VirTis BM 5000, SP Scientific, Warminster, PA, USA).

### 3.2. Supercritical Carbon Dioxide (SC-CO_2_) Extraction

Freeze-dried dabai pulp was ground together to produce no less than 0.2 mm powder. The ground dabai pulp powder was subjected to a large-scale SC-CO_2_ extraction at supercritical fluid center (SFC) Universiti Putra Malaysia. The supercritical fluid extraction using a carbon dioxide system was performed at a pressure of 40 MPa and a temperature of 40 °C. The extraction protocol was executed as described in detail from the method in our previous study [48]. DPO was collected in an opaque white plastic bottle and stored at 4 °C. The extraction process produced a waste known as DDP. The DDP was subsequently collected and stored at 4 °C.

### 3.3. DPO Extract Preparation and Phenolic Characterization

DPO (2 g) was placed into a polyethylene centrifuge tube (13 mL) containing 5 mL of 80% methanol (Thermo Fisher Scientific, Waltham, MA, USA). After vigorous shaking for 1 min by using a vortex, the sample was directly sonicated using POWERSONIC 405 (Hwashin Technology, Seoul, Korea) ultrasonic for 15 min. Later the sample was centrifuged at 5000 rpm for 25 min. The methanolic phase was then filtered through a 0.45 µm pore size and 17-mm diameter nylon filter [49]. All extracts were kept at 4 °C before further analysis.

Total phenolic content of DPO was evaluated by using the Folin Ciocalteu’s method, and the result was expressed as mg gallic acid equivalent (GAE) per g extract (mg GAE/g extract) [50].

Syringic acid in DPO was analyzed by high-performance liquid chromatography (HPLC, Agilent Technologies, Waldbronn, Germany). Chromatographic conditions used for HPLC analysis were modified based on a method described by Schneider [51]. An Agilent 1100 series (Agilent Technologies, Waldbronn, Germany) chromatograph equipped with a diode-array detector (DAD) was used. The solvent gradient (Solvent A: Water–Methanol–Acetic acid; 93:5:2 *v*/*v*/*v*, Solvent B: Methanol–Acetic acid; 98:2 *v*/*v*) (Thermo Fisher Scientific, MA, USA) was applied to a reversed-phase Lichrospher C-18 column (250 × 4 mm i.d., 5 μm) (Merck KGaA, Darmstadt, Germany) as follows: 0 min, 96% A; 10 min, 50% A; 15 min, 40% A; 30 min, 0% A. The flow rate was 1 mL min^−1^, and the column temperature was 20 °C. The volume injected was 20 μL. Quantification of syringic acid was carried out using the area values measurements at 280 nm. Identification of chromatographic peaks was carried out by comparing their retention times and spectra with those of standard. Quantitative assays were achieved using external calibration curves for standard phenols; syringic acid: y = 42.155x − 0.1654 (R = 1) (ChemFace, Wuhan, China).

### 3.4. DDP Extract Preparation and Phenolic Characterization

DDP (0.5 g) was placed into a polyethylene centrifuge tube (13 mL) containing 5 mL of 62.25% methanol (Thermo Fisher Scientific, Waltham, MA, USA). The concentration of methanol used in this extraction was previously optimized by Khoo et al. [29] for obtaining maximum levels of total phenolics. After vigorous shaking for 1 min by using a vortex, the sample was directly sonicated using POWER SONIC 405 (Hwashin Technology, Seoul, Korea) ultrasonic for 15 min. Later the sample was centrifuged at 5000 rpm for 25 min. The methanolic phase was then filtered through a 0.45 µm pore size and 17-mm diameter nylon filter [49]. All of the extracts were kept at 4 °C before further analysis.

Total phenolic content of DDP was evaluated by using the Folin Ciocalteu’s method, and the result was expressed as mg gallic acid equivalent (GAE) per g extract (mg GAE/g extract) [50]. The monomeric anthocyanin content (TAC) of DDP components was determined using a spectrophotometric pH differential protocol. The TAC in DDP was expressed as mg/100 g extract DDP [52].

Syringic acid in DDP was analyzed by high-performance liquid chromatography (HPLC, Agilent Technologies, Waldbronn, Germany). Chromatographic conditions used for HPLC analysis were modified based on a method described by Khoo et al. [53]. An Agilent 1100 series (Agilent Technologies, Waldbronn, Germany) chromatograph equipped with a diode-array detector (DAD) was used. The solvent gradient (Solvent A: Water–Methanol–Acetic acid; 93:5:2 *v*/*v*/*v*, Solvent B: Methanol–Acetic acid; 98:2 *v*/*v*) (Thermo Fisher Scientific, Waltham, MA, USA) was applied to a reversed-phase Lichrospher C-18 column (250 × 4 mm i.d., 5 μm) (Merck KGaA, Darmstadt, Germany) as follows: 0 min, 100% A; 30 min, 60% A; 32 min, 100% A. The flow rate was 1 mL min^−1^, and the column temperature was 30 °C. The volume injected was 20 μL. Quantification of syringic acid was carried out using the area values measurements at 280 nm. Identification of chromatographic peaks was carried out by comparing their retention times and spectra with those of standard. Quantitative assays were achieved using external calibration curves for standard phenols; syringic acid: y = 42.174x − 6.991 (R = 1) (ChemFace, Wuhan, China).

### 3.5. Experimental Diets Extract Preparation and Phenolic Characterization

A freeze-dried powdered experimental diet (0.1 g) was placed in a polyethylene centrifuge tube (13 mL). Later, 1 mL of 80% methanol (Thermo Fisher Scientific, Waltham, MA, USA) was added to the tube. The tube containing the DDP mixture was sonicated for 15 min. (POWERSONIC 405 Hwashin Technology, Seoul, Korea). The mixture was then vortexed each for 30 s and centrifuged for 5 min at 5000 rpm. The DDP extract was stored at −20 °C until further analysis [49].

Total phenolic content of DPO was evaluated by using the Folin Ciocalteu’s method, and the result was expressed as mg gallic acid equivalent (GAE) per g extract (mg GAE/g extract) [50].

### 3.6. Experimental Diets

#### 3.6.1. Normal Diet

The normal diet was prepared by properly mixing 18% corn starch, 50% sucrose, 12% casein, 5% cellulose, 3.5% mineral mixture, 1% vitamin mixture, 0.3% DL-methionine, 0.2% choline, 2% corn oil, and 8% ghee. The ingredients were mixed carefully, gently pressed on baking pans, cut into smaller pieces, and baked in an oven (Binder ED23, Tuttlingen, Germany) at 50 °C–60 °C for 24 h [54].

#### 3.6.2. High Cholesterol Diet

The high cholesterol diets were prepared by mixing 17% corn starch, 50% sucrose, 12% casein, 5% cellulose, 3.5% mineral mixture, 1% vitamin mixture, 0.3% DL-methionine, 0.2% choline, 2% corn oil, 8% ghee, and 1% cholesterol. The protocol for diet preparation and oven condition was like a normal diet.

#### 3.6.3. Treatment Diet

Treatment diets were prepared similarly to a high cholesterol diet. However, in the DPO treatment diet, 2% of corn oil was replaced with 2% dabai pulp oil. Meanwhile, in the DDP treatment diet, 2% of cellulose was replaced with 2% defatted dabai pulp.

### 3.7. Animals Study Design

#### 3.7.1. Acclimatization Period

All experiment protocols and ethical aspects were carefully followed and performed following the proper use and care of laboratory animals, as approved by the Institutional Animal Care and Use Committee (IACUC), Universiti Putra Malaysia (IACUC R045/2015, 30 September 2016). We purchased Male-specific pathogen-free (SPF) Sprague-Dawley rats (*n* = 30) at the age of 4 weeks, weighing between 100 to 150 g from Nomura Siam International Co., Ltd., Bangkok, Thailand. The rats were acclimatized for two weeks under individual ventilated cages (IVC) at Comparative Medicine and Technology Unit (COMeT) Universiti Putra Malaysia with the condition between 21 and 23 °C, at relative humidity in a range of 50 to 60% with regular light and dark cycle and free access to food and water.

#### 3.7.2. Hypercholesterolemic Induction Period

The rats were randomly divided into normal rats group (NG; *n* = 6), which received cholesterol-free diet (ND), and hypercholesterolemic rats group (*n* = 24) which received a high cholesterol diet containing 1% cholesterol (HC). After 30 days, all experimental rats fasted overnight. Blood (1 mL) was collected via cardiac puncture after the rats were intraperitoneally anaesthetized with ketamine (50 mg/kg body weight) and xylazine (10 mg/kg body weight) for hypercholesterolemia screening. The blood was withdrawn slowly to prevent the heart from collapsing. Rats with total serum cholesterol and LDL-C significantly higher than NG rats were considered hypercholesterolemia rats [55].

#### 3.7.3. DPO and DDP Treatment Period

Hypercholesterolemic rats group (*n* = 24) were further randomized into four groups: (1) Hypercholesterolemic positive control rats group (PG) (*n* = 6) continuously fed on high cholesterol diet (HC); (2) hypercholesterolemic rats treated with 2% DPO (HG) (*n* = 6) fed with DPO treatment diet; (3) hypercholesterolemic rats treated with 2% DDP (DG) (*n* = 6) fed with DDP treatment diet; and lastly (4) hypercholesterolemic rats treated with 10 mg/kg simvastatin (SG) (*n* = 6) continuously fed on HC. The treatment period lasts for 30 days. On the last day of the treatment period, the rats fasted for 16 hr. Later, the rats were intraperitoneally anaesthetized with ketamine (50 mg/kg body weight) and xylazine (10 mg/kg body weight) by a veterinarian. Blood (3 mL) was collected into sterile tubes via cardiac puncture before euthanasia by exsanguination via a cardiac puncture through the heart’s abdominal aorta. Further, the liver was harvested for histology evaluation.

### 3.8. Biochemistry Evaluation

Blood was collected in a sterile tube. The blood was centrifuged at 3000 rpm at 4 °C for 10 min, and the serum was stored at −80 °C. Serum lipid profile was measured by using Dimension^®^ Xpand^®^ Plus (Siemens Healthcare Diagnostics, Newark, DE, USA). For each parameter, the evaluations were conducted based on manufacturer instructions; total cholesterol (Siemens Healthcare, DF27), triglyceride (Siemens Healthcare, DF69A), low-density lipoprotein (Siemens Healthcare, DF131), and high-density lipoprotein (Siemens Healthcare, DF48B). The HMG-CoA reductase was evaluated using a rat HMG-CoA ELISA kit (Wuhan Fine Biotech Co., Ltd., Wuhan, China). All procedures were conducted attentively and precisely according to the manufacturer’s instructions.

### 3.9. Liver Histology Evaluation

Toxicity profile (AST and ALT) was evaluated using BioLis 24i Premium Chemistry Analyzer (BioRex Mannheim Malaysia Sdn. Bhd, Petaling Jaya, Malaysia). For each parameter, the evaluations were conducted based on manufacturer instructions; AST (aspartate aminotransferase EC.2.6.11 according to International Federation of Clinical Chemistry and Laboratory Medicine [IFCC]) and ALT (alanine aminotransferase EC.2.6.11 according to IFCC). All procedures were conducted attentively and precisely according to the manufacturer’s instructions.

After sacrificing the rats, the liver was harvested and rinsed in saline. The organ was carefully patted between paper towels and weighed using an electronic scale balance (Shimadzu BL3200HL, Tokyo, Japan) then kept in a urine container (10% neutral buffered formalin). During the day of liver histology slide preparation, the liver was embedded in paraffin wax. Next, the liver section was sectioned into 5 µm thickness using a microtome (Leica Biosystems RM2155, Wetzlar, Germany). Later, the sectioned liver was mounted on a microscope slide, deparaffinized with xylene, and then rehydrated with ethanol solutions (100%, 90%, and 70%, respectively) (Thermo Fisher Scientific, Waltham, MA, USA). Then, the slides were stained with hematoxylin and eosin. The prepared liver histology slides were covered with a coverslip and observed under a light microscope (Motic BA410, Xiamen, China) equipped with a digital camera (Moticam Pro, Xiamen, China) at 200× magnification. The image was taken and processed using Motic Images Plus 2.0 software (Motic, Xiamen, China). Five random, non-overlapping fields were selected from each slide. A representative picture was randomly selected from each group. The relative organ weight (ROW) was calculated as follows:

ROW = [organ weight (g)/Final body weight of rat on sacrifice day (g)] × 100(1)

### 3.10. Statistical Analysis

Data were expressed as mean ± standard deviation (*n* = 6). Data were analyzed by using one-way ANOVA using SPSS for windows version 23, International Business Machines Corporation (IBM), Armonk, NY, USA. Duncan’s multiple range test was used to test whether there were significant differences among the experimental groups. Values were considered statistically significant when *p* < 0.05

## 4. Conclusions

In conclusion, this study provides evidence that DDP shows superior activity against hypercholesterolemia by significantly reducing total serum cholesterol, triglyceride, LDL-C, and HMG-CoA-r levels. DPO and DDP extracts contain syringic acid, which offers hepatoprotective activity in the high-cholesterol diet-fed rat. These results suggest potential DPO and DDP applications for functional properties in foods rich with the bioactive compound. Supercritical carbon dioxide extraction is a novel technique applied to produce non-toxic DPO and DDP. Future research is needed to verify the effectiveness of DDP and DPO against hypercholesterolemia disease progression. This information is valuable to get acquainted with DDP and DPO’s practical value as functional foods in the future.

## Figures and Tables

**Table 1:** Total phenolic content in the experimental diet.

Experimental Diet	Total Phenolic Content (mg GAE/g Extract)
ND	2.842 ± 0.12 ^c^
HC	2.649 ± 0.05 ^d^
2% DPO	3.115 ± 0.00 ^b^
2% DDP	3.969 ± 0.01 ^a^

Values are the mean ± standard deviation (*n* = 3). Different letters indicate significant differences (*p* < 0.05) between groups by Duncan’s multiple range tests. ND: normal diet; HC: high cholesterol diet; 2% DPO: 2% dabai pulp oil diet; 2% DDP: 2% defatted dabai pulp diet.

**Table 2:** Beneficial effects of DPO and DDP on biochemistry profile in hypercholesterolemic rats.

Group	TC (mmol/L)	TG (mmol/L)	LDL-C (mmol/L)	HDL-C (mmol/L)	HMG-CoA-r (ng/mL)
NG	1.57 ± 0.15 ^a^	1.97 ± 0.92	0.17 ± 0.06 ^a^	1.36 ± 0.14	1.47 ± 0.07 ^a^
PG	2.12 ± 0.65 ^b^	2.08 ± 0.65	0.50 ± 0.19 ^b^	1.27 ± 0.53	2.02 ± 0.24 ^b^
HG	1.51 ± 0.18 ^a^	1.10 ± 0.30 ^ab^	0.42 ± 0.13 ^b^	1.22 ± 0.16	1.64 ± 0.12 ^ab^
DG	1.37 ± 0.25 ^a^	1.18 ± 0.38 ^ab^	0.33 ± 0.11 ^ab^	1.25 ± 0.19	1.43 ± 0.07 ^a^
SG	1.23 ± 0.05 ^a^	1.49 ± 0.28	0.24 ± 0.05 ^a^	1.08 ± 0.07 ^b^	1.39 ± 0.04 ^a^

TC, total cholesterol; TG, triglyceride; LDL-C: low-density lipoprotein cholesterol; HDL-C: high-density lipoprotein cholesterol; HMG-CoA-r: 3-hydroxy-3-methyl-glutaryl-CoA reductase; NG, normal rats group; PG, hypercholesterolemic positive control rats group; HG, hypercholesterolemic rats treated with 2 % DPO; DG, hypercholesterolemic rats treated with 2 % DDP; SG, hypercholesterolemic rats treated with 10 mg/kg simvastatin. ^a^ Indicates a statistically significant difference (*p* < 0.05) versus PG group; ^b^ indicates a statistically significant difference (*p* < 0.05) versus NG group by Duncan’s multiple range tests using SPSS for windows version 23. Results are given as man ± SD (*n* = 6).

**Table 3:** Histopathological changes and liver weight in rats treated with DPO and DDP.

Group	AST (U/L)	ALT (U/L)	Liver Weight (g/100 g BW)	Histopathological Changes	
				Hepatic Steatosis ^1^	
				Microvesicular	Macrovesicular
NG	81.83 ± 4.17 ^a^	20.33 ± 3.14 ^a^	3.73 ± 0.32 ^a^	-	-
PG	124.33 ± 23.90 ^b^	30.33 ± 7.66 ^b^	4.64 ± 0.46 ^b^	+++	+++
HG	101.33 ± 18.54 ^a^	23.17 ± 4.26 ^a^	3.95 ± 0.39 ^a^	++	++
DG	88.83 ± 13.73 ^a^	22.33 ± 3.01 ^a^	3.83 ± 0.28 ^a^	++	++
SG	105.67 ± 15.74 ^b^	33.83 ± 4.88 ^b^	4.16 ± 0.48 ^a^	++	++

NG, normal rats group; PG, hypercholesterolemic positive control rats group; HG, hypercholesterolemic rats treated with 2% DPO; DG, hypercholesterolemic rats treated with 2% DDP; SG, hypercholesterolemic rats treated with 10 mg/kg simvastatin. ^a^ Indicates a statistically significant difference (*p* < 0.05) versus PG group; ^b^ indicate a statistically significant difference (*p* < 0.05) versus NG group by Duncan’s multiple range tests using SPSS for windows version 23. Results are given as mean ± SD (*n* = 6). ^1^ The severity was evaluated based on the following scoring scheme: - normal, + mild effect (<33%), ++ moderate effect (33% to 66%), +++ severe effect (>66%).

## Data Availability

The data presented in this study are available on request from the corresponding author.

## Sample Availability

Not available.
